# Association of American Medical Colleges

**Published:** 1882-01-20

**Authors:** 


*ASSOCIATION OF AMERICAN MEDICAL COLLEGES.*

Some very hard things are now being said of the American Medical Colleges. To instance the subjoined remarks in "Gillard's Medical Journal," copied in the St. Louis "Clinical Record," and indorsed by the editor of that Journal as a correct report of its history.

We copy the extract without further comment than to say that we never abused the Association, although we did not regard the plans adopted as the best to accomplish the important objects sought. We always favored, and still favor higher education, and have urged our brethren of the press to unite their efforts in encouraging the profession to this end. It was the design of the College with which we are connected to unite with the Association, but when we became aware that the pledges assumed by its members were not in every instance faithfully observed, even by some who boasted of their connection with it—using it to the disparagement of the nonaffiliated schools, among which are a number of the best institutions in the country—we hesitated and finally declined to make the connection.

The following is the extract alluded to:

"The readers of this journal (Clinical Record) are well aware of its long entertained doubt as to the reliability and efficiency of this organization. It was organized chiefly through the action of the Jefferson Medical College of Philadelphia, which, though receiving from year to year the honor of its presidency, has conspicuously refused to sustain it. It was god-fathered by the College of Physicians and Surgeons of New York, which has long since abandoned it. And it was dry-nursed awhile by Bellevue, which has repudiated it.

"Through several years of unsteady and feeble existence, it has tottered along, repudiated by many, ridiculed by more, and distrusted even by its alleged supporters. Its downfall has been predicted by this journal almost as often as its inefficiency has been demonstrated. And now what is the last manifestation of its vitality, reliability and genuineness? It met once at Richmond, Va., when the Jefferson Medical College declined any longer the honor of its Presidency!! New officers, Dr. J. M. Bodine, of Kentucky, President; Dr. Briggs, of Nashville, Vice-President, and Dr. Leartus Connor, Secretary (all excellent gentlemen), were elected; it then hopelessly adjourned, and never succeeded again in obtaining a quorum; and the last abortive effort at a meeting was adjourned subject to the call of the President!! When one remembers what this noisy and curious little body promised to do, what its fulminatioin and resolutions and fuss have been, and reads now of the pitiable result, he can only exclaim, *montes parturiunt et nacitur ridiculus mus !*"

